# Pan-Family Analysis of HAK/KUP/KT Potassium Transporters in *Brassica napus* Prioritizes a Candidate Locus Associated with Salt-Related Variation

**DOI:** 10.3390/genes17080893

**Published:** 2026-07-29

**Authors:** Mingxuan Yao, Yuhao Chu, Xiaokang Dai

**Affiliations:** Engineering Research Center of Agricultural Microbiology Technology, Ministry of Education & Heilongjiang Provincial Key Laboratory of Ecological Restoration and Resource Utilization for Cold Region & Key Laboratory of Microbiology, College of Heilongjiang Province & School of Life Sciences, Heilongjiang University, Harbin 150080, China

**Keywords:** *Brassica napus*, HAK/KUP/KT, pan-family analysis, potassium transporter, salt-associated variation, candidate prioritization

## Abstract

The HAK/KUP/KT family represents a major group of plant potassium transporters involved in K^+^ uptake, ion homeostasis and stress responses. However, the accession-level diversity of *HAK/KUP/KT* genes in *Brassica napus* remains insufficiently characterized. In this study, we performed a pan-family analysis of *HAK/KUP/KT* genes across eight *B. napus* accessions. A total of 269 annotated HAK/KUP/KT family members were identified and classified into core, soft-core, dispensable and private orthogroups based on their representation across the analyzed genome annotations. Phylogenetic analysis grouped these proteins into four major clades together with reference HAK/KUP/KT members from *Arabidopsis thaliana* and rice. Ka/Ks analysis indicated that HAK/KUP/KT orthogroups were predominantly under purifying selection, while accession-variable orthogroups showed greater variation in sequence conservation. Gene structure, conserved domain, motif and predicted promoter cis-element analyses revealed conserved transporter-related protein features together with orthogroup-level structural and sequence variation. Expression profiling using the ZS11 BnIR dataset further revealed tissue-, hormone- and stress-responsive expression patterns among ZS11 *HAK/KUP/KT* genes. By integrating expression features, predicted promoter information, evolutionary characteristics, published salt GWAS context and BnVIR haplotype–phenotype information, *BnaA08T0085800ZS* was prioritized as a candidate locus located near salt-associated variation. This study provides a pan-genome perspective on HAK/KUP/KT family diversity in *B. napus* and establishes a framework for prioritizing candidate genes for future functional investigation.

## 1. Introduction

*Brassica napus* L. is an important oilseed crop worldwide and provides a major source of edible vegetable oil and plant protein [1,2]. However, its growth, yield formation and geographic adaptation are frequently affected by abiotic stresses, among which soil salinity is a major limiting factor [3]. Salt stress can inhibit seed germination, restrict seedling growth, reduce photosynthetic capacity and disturb cellular ion balance [3,4]. In particular, excessive Na^+^ accumulation disrupts K^+^ uptake and homeostasis, leading to an unfavorable Na^+^/K^+^ ratio and impaired physiological performance under saline conditions [5,6]. Therefore, identifying ion transporter genes involved in potassium uptake and salt stress responses is important for understanding salt tolerance mechanisms and improving stress resilience in *B. napus* [5,7].

The *HAK/KUP/KT* family represents one of the major potassium transporter families in plants, and its members are involved in K^+^ uptake, long-distance K^+^ transport, ion homeostasis, plant development and stress responses [8,9]. This family has been systematically characterized in several representative species, including *Arabidopsis thaliana*, rice and barley, providing important references for phylogenetic classification and functional interpretation of plant *HAK/KUP/KT* genes [10,11,12]. Functional studies further support the role of *HAK/KUP/KT* members in K^+^ acquisition and stress adaptation. For example, barley *HAK1* encodes a high-affinity potassium transporter, while wheat *TaHAK13* is induced by drought, low-K^+^ and salt treatments [13,14]. In addition, salt tolerance is often accompanied by coordinated changes in ion transporter expression, ROS accumulation and antioxidant capacity, as shown in a recent study comparing salt responses between *Triticum aestivum* and *T. durum* [15]. In *B. napus*, a previous single reference genome survey identified 40 *BnaHAK* genes and suggested that several members may respond to K^+^ deficiency [16]. However, given the allotetraploid origin and extensive accession-level genomic variation in *B. napus*, a single reference genome may not fully represent the diversity of this gene family [17]. Therefore, pan-family analysis across multiple *B. napus* genomes is needed to distinguish conserved *HAK/KUP/KT* members from accession-variable orthogroups and to evaluate their evolutionary and stress-response divergence.

Recent pan-genome studies have revealed extensive gene content variation, copy number variation and presence–absence variation in *B. napus*, indicating that accession-level diversity cannot be fully represented by a single reference genome [17]. For gene families, pan-genome-based analysis provides a useful framework for separating conserved members from accession-variable genes. This strategy has been applied to several *B. napus* gene families, such as *SPL* and *SWEET*, where pan-family identification was combined with trait-related or abiotic stress expression analyses [18,19]. Similar pan-genome-wide strategies have also been used for transporter families in other plants, such as the ZIP family in cucumber [20]. These studies suggest that pan-family analysis can provide additional information beyond single reference gene identification, especially for multi-copy gene families potentially involved in stress adaptation. However, a pan-family-level analysis of *HAK/KUP/KT* potassium transporter genes in *B. napus* has not yet been systematically performed.

In this study, we performed a pan-family analysis of *HAK/KUP/KT* potassium transporter genes across eight *B. napus* accessions. A total of 269 annotated HAK/KUP/KT family members were retained from the eight published proteomes and clustered into orthogroups. Based on their representation across the eight accessions, these orthogroups were classified as core, soft-core, dispensable or private. To characterize their evolutionary and functional divergence, we integrated phylogenetic classification, chromosomal distribution of orthogroup members, *Ka/Ks* estimation, gene structure, conserved domain and motif analysis, promoter cis-element composition and expression profiling. In addition, published salt GWAS signals and BnVIR haplotype–phenotype evidence were incorporated to prioritize *HAK/KUP/KT* candidates potentially associated with salt-related variation. This study provides a pan-genome-level view of *HAK/KUP/KT* family diversity in *B. napus* and prioritizes the *BnaA08T0085800ZS* locus for future investigation of its possible contribution to potassium transport and salt-related phenotypic variation.

## 2. Materials and Methods

### 2.1. Genomic Resources and Identification of HAK/KUP/KT Genes

Genome assemblies and corresponding gene annotations of eight *B. napus* accessions, including ZS11, Gangan, No2127, Quinta, Shengli, Tapidor, Westar and Zheyou7, were obtained from the same pan-genome study [17], in which the eight accessions were processed using a common genome assembly and annotation framework. The corresponding protein sequences, coding sequences and GFF3 annotation files were used for HAK/KUP/KT gene identification and subsequent analyses. Previously characterized HAK/KUP/KT proteins from *Arabidopsis thaliana* [10] and rice (*Oryza sativa*) [9] were included as reference sequences for phylogenetic classification, and their sequences were retrieved according to gene IDs reported in earlier studies.

To ensure consistent family identification across accessions, the same HMM-based screening threshold, manual domain confirmation, incomplete-sequence filtering and isoform-selection criteria were independently applied to all eight published proteomes. The conserved K_trans domain (PF02705) was used to search the protein dataset of each accession using HMMER v3.3.2 [21], and proteins with an E-value ≤ 1 × 10^−10^ were retained as preliminary candidates. The retained candidate sequences were then manually inspected to confirm the presence and apparent completeness of the conserved K_trans domain, and sequences lacking the characteristic domain or showing obvious truncation or incomplete annotation were removed. For gene loci with multiple isoforms, only the longest protein isoform was retained to avoid redundancy. After domain confirmation and isoform filtering, 269 annotated HAK/KUP/KT family members were retained across the eight published proteomes. Basic information for each retained family member, including accession source, gene ID, chromosome location, strand, protein length and sub-genome assignment, was extracted from the corresponding genome annotation files. The final identified *HAK/KUP/KT* genes and their basic annotations are listed in Supplementary Table S1.

### 2.2. Orthogroup Clustering, Pan-Family Classification and Chromosomal Distribution

To assess the conservation and accession-level variation in HAK/KUP/KT genes, the non-redundant HAK/KUP/KT protein sequences identified from the eight *B. napus* accessions were clustered into orthogroups using OrthoFinder v2.5.5 [22]. The presence and copy number of each orthogroup were then summarized across the eight accessions. To generate the pan-family and core-family accumulation curves, all possible accession-addition orders were enumerated (8! = 40,320 permutations). For each order and each number of included accessions (k = 1–8), the number of pan orthogroups was defined as the number of orthogroups represented in at least one included accession, whereas the number of core orthogroups was defined as the number of orthogroups represented in all included accessions. For each value of k, the plotted points represent the mean numbers of pan and core orthogroups across all permutations, and the shaded regions represent the corresponding means ±1 standard deviation. According to the number of accessions in which each orthogroup was represented, HAK/KUP/KT orthogroups were classified into four pan-family categories: core, represented in all eight accessions; soft-core, represented in six or seven accessions; dispensable, represented in two to five accessions; and private, represented in only one accession. The soft-core category was operationally defined as orthogroups represented in at least 75% but fewer than 100% of the analyzed accessions, thereby distinguishing broadly represented but non-universal orthogroups from those represented in fewer accessions. For gene-level summaries, each individual gene was annotated according to the pan-family category of the orthogroup to which it belonged. For each accession, the total number of HAK/KUP/KT genes and the numbers of genes belonging to core, soft-core, dispensable and private orthogroups were calculated. Orthogroup membership, accession-level copy number and pan-family classification are summarized in Supplementary Table S2.

Genomic positions of HAK/KUP/KT genes were extracted from the corresponding GFF3 annotation files, and genes were assigned to the A or C sub-genome according to their chromosome labels. To visualize the chromosomal distribution of HAK/KUP/KT members across accessions, genes were plotted according to their annotated chromosomal positions, and genes assigned to the same OrthoFinder orthogroup were connected. These links indicate shared orthogroup membership only and were not interpreted as evidence of synteny, conserved genomic position, or specific gene loss, duplication or expansion events.

### 2.3. Phylogenetic Analysis and Ka/Ks Estimation

For phylogenetic analysis, the 269 HAK/KUP/KT protein sequences identified from the eight *B. napus* accessions were combined with 13 previously characterized HAK/KUP/KT proteins from *Arabidopsis thaliana* and 27 HAK/KUP/KT proteins from rice. The reference protein names and gene identifiers are provided in Supplementary Table S7. Full-length amino acid sequences were aligned using MAFFT v7 with the auto strategy and otherwise default settings [23]. Poorly aligned and gap-rich positions were removed using trimAl v1.4.rev15 with the -automated1 option. A maximum-likelihood phylogenetic tree was reconstructed using IQ-TREE 2 under the LG+F+G4 amino acid substitution model [24]. Branch support was evaluated using 1000 ultrafast bootstrap replicates. Because no external outgroup outside the HAK/KUP/KT family was included, the tree was treated as unrooted. The tree was visualized and annotated using iTOL v6 [25], with accession, species, orthogroup membership and orthogroup pan-status displayed as annotation tracks.

To evaluate evolutionary constraints among *HAK/KUP/KT* orthogroups, pairwise *Ka/Ks* values were calculated using the coding sequences of genes within the same orthogroup. Orthogroups containing at least two coding sequences were included in this analysis. For each gene pair, protein sequences were first aligned using MAFFT [23], and the corresponding CDS sequences were then converted into codon-based alignments according to the protein alignments using PAL2NAL v14 [26]. *Nonsynonymous substitution rate (Ka), synonymous substitution rate (Ks)* and *Ka/Ks* ratio were estimated using KaKs_Calculator v2.0 [27] with the model-averaging method. Pairwise comparisons were excluded if codon alignment failed, Ka or Ks values were missing or non-finite, Ka was negative, or Ks was less than or equal to zero. Pairwise *Ka/Ks* estimates were summarized descriptively at the orthogroup and pan-status levels. Because multiple pairwise comparisons within the same orthogroup share sequences and are therefore not statistically independent, the distributions among pan-status categories were visualized for descriptive purposes only. No formal statistical tests treating individual gene pairs as independent observations were performed in the revised analysis.

### 2.4. Gene Structure, Conserved Domain, Transmembrane Topology, Meme Motif and Promoter Cis-Element Analysis

Multi-member HAK/KUP/KT orthogroups were selected to provide illustrative examples from the core, soft-core and dispensable categories and to permit comparison of annotated gene and protein structures across accessions. Private orthogroups were not included because they lacked sufficient cross-accession members for within-orthogroup structural comparison. The selected orthogroups were treated as illustrative examples rather than as statistically representative samples of the entire family.

Gene structure information, including exon, intron and CDS positions, was extracted from the corresponding GFF3 annotation files. Gene structures were displayed according to their actual genomic lengths to retain differences in gene size among orthogroup members. Conserved domain positions were determined based on the K_trans domain annotation of *HAK/KUP/KT* proteins. Protein sequences included in Figure 4 were analyzed together using MEME Suite v5.5.8 [28] in protein mode. The zero-or-one occurrence per sequence model (zoops) was used, with a maximum of 10 motifs requested and allowable motif widths ranging from 6 to 50 amino acids. All other parameters were left at their default settings. The identified motif positions and K_trans domain regions were mapped onto the corresponding protein sequences and visualized according to actual protein length. These analyses were used to describe K_trans domain positions and MEME motif patterns among the selected orthogroups. Motif differences involving unusually long or short predicted proteins were interpreted descriptively because gene model fusion, fragmentation or incomplete annotation could not be excluded. Detailed gene structure, domain and motif information is provided in Supplementary Table S3. Transmembrane topology was predicted for all 269 identified HAK/KUP/KT proteins using DeepTMHMM v1.0 with default settings. The predicted topology class, number of transmembrane helices and helix coordinates for each protein are provided in Supplementary Table S8. These predictions were used to describe protein topology but were not regarded as definitive validation of the corresponding gene models.

For promoter analysis, the available upstream sequence extending up to 2 kb from the annotated translation start site was extracted according to gene orientation. To reduce biases caused by incomplete or overlapping upstream regions, promoter extraction was truncated at the boundary of the nearest upstream annotated gene, chromosome or scaffold boundary, or unresolved sequence gap. The actual length of each extracted promoter sequence was recorded, and promoters containing unresolved sequence gaps were flagged in Supplementary Table S4. Cis-acting regulatory elements were predicted using PlantCARE [29] and classified into ABA-, auxin-, GA-, JA-, stress-, light- and development-related categories according to their database annotations. Because PlantCARE motifs are common sequence features and their presence does not demonstrate regulatory activity, the predictions were used only as descriptive promoter annotations. Raw motif counts were not interpreted as evidence of regulatory enrichment or regulatory potential, and no inferential comparison among pan-status categories was performed. The promoter cis-element results are provided in Supplementary Table S4.

### 2.5. Expression Profiling and Integrated Candidate Scoring

Because the BnIR expression data used here were derived exclusively from the ZS11 accession, this analysis was intended to describe expression patterns among ZS11 HAK/KUP/KT genes rather than expression divergence among the eight accessions. Expression profiles of ZS11 *HAK/KUP/KT* genes were obtained from the BnIR expression profile database (ZS11 library) (https://yanglab.hzau.edu.cn/BnIR/expression_zs11 (accessed on 25 May 2026)) [30]. Hormone- and stress-treatment expression datasets were downloaded from the BnIR ZS11 expression module and matched to the identified ZS11 *HAK/KUP/KT* gene set according to gene identifiers. To ensure consistency between datasets, gene IDs were standardized before downstream analysis. The database-reported TPM values were standardized for each gene using row-wise Z-score transformation and visualized as heatmaps. The resulting Z-scores were used only to describe the relative expression pattern of each gene across the selected ZS11 tissues and treatments and were not interpreted as absolute expression levels or statistically significant differential expression. Genes were annotated with their orthogroup, phylogenetic clade and pan-status information.

To prioritize candidate HAK/KUP/KT genes potentially involved in stress-related responses, descriptive expression features were summarized for each ZS11 member, including basal expression level, maximum expression across hormone treatments, maximum expression across abiotic stress treatments and expression variation across treatment samples. Predicted promoter cis-element composition, pan-family status and orthogroup conservation were incorporated as supporting features in the exploratory candidate-prioritization analysis. The promoter-derived component was treated as ancillary predicted sequence information rather than as independent evidence of regulatory activity or regulatory enrichment. Each candidate feature was normalized to a 0–1 range using min–max normalization across the 41 ZS11 HAK/KUP/KT genes. Related features were grouped into expression, predicted promoter and conservation components, and the three components were assigned equal weights when calculating the integrated score; pan-status and orthogroup conservation were combined within the conservation component to reduce double counting. Within each evidence category, the normalized feature values were averaged to obtain the corresponding component score, and the final integrated score was calculated as (E+P+C)/3, where E, P and C represent the expression, predicted promoter and conservation component scores, respectively. Genes were ranked according to the resulting continuous score, and the highest-ranking genes were retained as exploratory candidates for further interpretation rather than being classified using a fixed functional cut-off. Detailed feature values and integrated candidate scores are provided in Supplementary Table S5.

### 2.6. Integration of Published Salt Gwas and Bnvir Haplotype Evidence

To evaluate whether the prioritized HAK/KUP/KT candidate was associated with genomic regions previously linked to salt-related traits, significant SNPs, associated traits and P values were obtained from the supplementary GWAS dataset reported by Zhang et al. [7]. The original GWAS SNP coordinates were based on the Darmor-bzh v4.1 reference genome, and candidate regions were defined within 200 kb upstream and downstream of lead SNPs. Because the HAK/KUP/KT genes in the present study were annotated in the ZS11 genome and no genome-wide coordinate conversion between the two assemblies was performed, exact physical distances between published GWAS SNPs and ZS11 gene models were not treated as definitive evidence. The published GWAS signals were therefore used only as locus-level contextual information.

The lead salt-associated SNPs, associated traits, reported *p* values and corresponding candidate intervals were summarized descriptively. Multiple significant SNPs within the same local interval were considered potentially linked markers rather than independent association signals. No composite score combining SNP number, physical distance and *p* value was calculated, and no new GWAS or linkage-disequilibrium analysis was performed. The published GWAS evidence was used as complementary locus-level information rather than as evidence that *BnaA08T0085800ZS* was the causal gene.

For the prioritized candidate *BnaA08T0085800ZS*, variant and haplotype information were obtained from BnVIR [31] using the corresponding G-type identifier BnaA08G0085800ZS. SNP distribution, haplotype structure, haplotype frequency and salt-related phenotype distributions among major haplotypes were examined based on BnVIR records. The BnVIR-derived haplotype–phenotype information was used as an additional description of variation at the candidate locus. Because the extent of accession and phenotype overlap between BnVIR and the published GWAS population was not established, the BnVIR and GWAS results were not treated as independent lines of evidence. Haplotype group sizes were highly unequal, and the database-derived phenotype comparisons were not reanalyzed using population structure, kinship, environmental covariates or multiple-testing correction because the required individual-level data were not available. The BnVIR results were therefore treated as descriptive haplotype–phenotype patterns rather than as formal tests of haplotype effects. The published salt GWAS information and BnVIR variant and haplotype evidence are provided in Supplementary Table S6.

### 2.7. RNA Extraction and qRT-PCR Analysis

ZS11 seedlings were treated with 200 mM NaCl, and leaf tissues were collected at 0, 1, 6 and 12 h after treatment, with the 0 h sample used as the control. Total RNA was extracted using a commercial plant RNA extraction kit according to the manufacturer’s instructions. First-strand cDNA was synthesized using a commercial reverse-transcription kit. Quantitative real-time PCR was performed using SYBR Green chemistry on a real-time PCR detection system. The amplification program consisted of 95 °C for 30 s, followed by 40 cycles of 95 °C for 5 s and 60 °C for 30 s, followed by melting curve analysis. The *B. napus GAPDH* gene was used as the internal reference. Gene-specific primers for *BnaA08T0085800ZS* were designed using Primer-BLAST (v2.5.0) and checked against the ZS11 genome and closely related homeologues. Primer specificity was further confirmed by the presence of a single melting curve peak. Four independent biological replicates and three technical replicates were included for each time point. Relative expression levels were calculated using the 2^−ΔΔCt^ method. Statistical differences among time points were evaluated by one-way ANOVA followed by Tukey’s multiple-comparison test.

## 3. Results

### 3.1. Genome-Wide Identification and Pan-Family Composition of HAK/KUP/KT Genes

**Figure 1:**
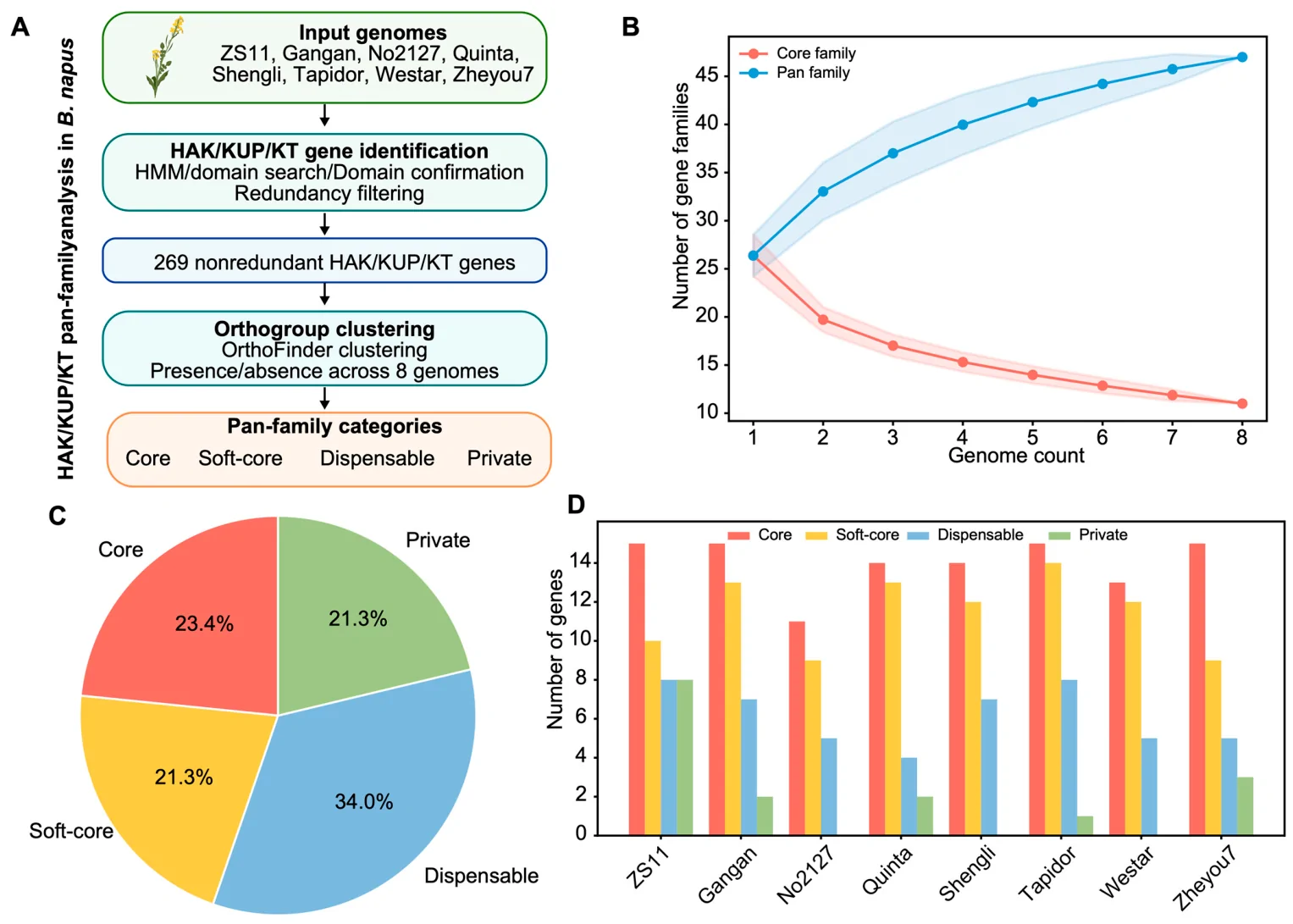
Overview of HAK/KUP/KT pan-family identification in eight *B. napus* accessions. (**A**) Schematic workflow showing HAK/KUP/KT gene identification, orthogroup clustering and pan-family classification. Candidate genes were identified from eight *B. napus* genomes by HMM/domain search, domain confirmation and redundancy filtering. (**B**) Pan-family and core-family accumulation curves generated from all 40,320 possible accession-addition orders. For each number of included accessions, the points indicate the mean numbers of pan and core orthogroups across all permutations, and the shaded regions indicate ±1 standard deviation. Pan orthogroups were defined as those represented in at least one included accession, whereas core orthogroups were represented in all included accessions. (**C**) Percentage distribution of HAK/KUP/KT orthogroups among the core, soft-core, dispensable and private categories. Orthogroups are the counting unit in this panel. (**D**) Numbers of individual HAK/KUP/KT genes in each accession, grouped according to the pan-family category of the orthogroup to which each gene belongs. Genes, rather than orthogroups, are the counting unit in this panel.

Genome-wide identification of *HAK/KUP/KT* potassium transporter genes was performed across eight *B. napus* accessions, including ZS11, Gangan, No2127, Quinta, Shengli, Tapidor, Westar and Zheyou7. After domain confirmation and redundancy filtering, 269 non-redundant *HAK/KUP/KT* genes were identified (Figure 1A), with HAK/KUP/KT members detected in all eight analyzed proteomes. The number of HAK/KUP/KT members varied among accessions, ranging from 25 in No2127 to 41 in ZS11, showing accession-level variation in the number of identified family members.

Orthogroup-based pan-family analysis revealed both conserved and variable components within the *HAK/KUP/KT* family. Accumulation analysis across all 40,320 possible accession-addition orders showed that the mean number of pan orthogroups increased progressively as additional accessions were included, whereas the mean number of orthogroups shared by all included accessions decreased (Figure 1B). The rates of change became smaller when six to eight accessions were included, indicating that the detected HAK/KUP/KT pan-family was approaching, although not necessarily reaching, saturation within the analyzed eight-accession panel. These results also indicate that no single accession captured all HAK/KUP/KT orthogroups detected across the eight accessions. Based on presence–absence patterns across the eight accessions, *HAK/KUP/KT* orthogroups were classified into core, soft-core, dispensable and private categories. At the orthogroup level, core, soft-core, dispensable and private gene families accounted for 23.4%, 21.3%, 34.0% and 21.3% of the *HAK/KUP/KT* orthogroups, respectively (Figure 1C). Thus, accession-variable orthogroups, especially dispensable and private categories, constituted a substantial proportion of the *HAK/KUP/KT* pan-family.

At the accession level, each genome contained genes belonging to core, soft-core and dispensable orthogroups, whereas the numbers of genes belonging to private orthogroups differed among accessions (Figure 1D). By definition, each core orthogroup was represented in all eight accessions, although the number of member genes within a given core orthogroup could differ among accessions. Figure 1D therefore summarizes individual gene counts grouped according to the pan-family category of their corresponding orthogroups. Genes belonging to dispensable and private orthogroups contributed to accession-level differences in the total number of HAK/KUP/KT genes.

### 3.2. Phylogenetic Classification, Chromosomal Distribution and Evolutionary Constraints

**Figure 2:**
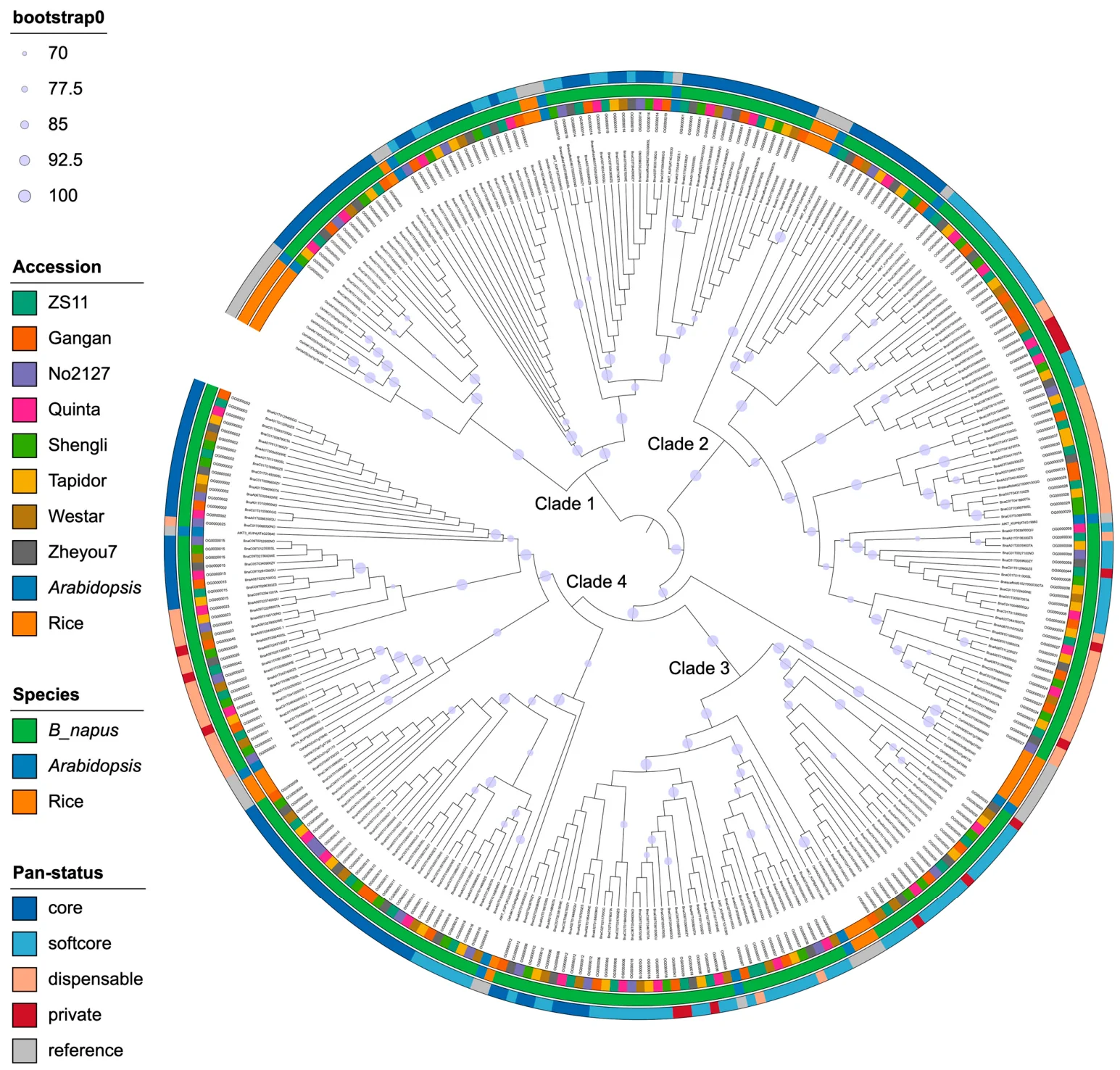
Phylogenetic classification of HAK/KUP/KT proteins from eight *B. napus* accessions, *Arabidopsis* and rice. A maximum-likelihood phylogenetic tree was constructed using the identified HAK/KUP/KT proteins from eight *B. napus* accessions together with reference HAK/KUP/KT members from *Arabidopsis* and rice. The tree classified the HAK/KUP/KT proteins into four major clades. External annotation tracks indicate species or accession source, orthogroup membership and the pan-family category of the orthogroup to which each gene belongs. Node size indicates branch support. Reference members from *Arabidopsis* and rice were included to assist clade assignment and provide phylogenetic context for the *B. napus HAK/KUP/KT* genes. Phylogenetic proximity to these reference proteins was not interpreted as evidence of one-to-one orthology or functional equivalence.

To investigate the evolutionary relationships of *HAK/KUP/KT* genes in *B. napus*, the identified proteins were analyzed together with previously characterized *HAK/KUP/KT* members from *Arabidopsis* and rice. These two species have been widely used as references for plant *HAK/KUP/KT* family classification, and their *HAK/KUP/KT* members have been systematically characterized in previous studies [9,10]. The maximum-likelihood phylogenetic tree divided the *HAK/KUP/KT* proteins into four major clades, broadly consistent with previous classifications of plant *HAK/KUP/KT* transporters [16,32]. *B. napus* members were distributed across all four clades together with Arabidopsis and rice reference proteins, supporting their classification as *HAK/KUP/KT* family members (Figure 2). The distribution of orthogroup pan-status categories on the tree showed that genes belonging to core, soft-core and dispensable orthogroups occurred in multiple clades, indicating that accession-variable orthogroups were not restricted to a single evolutionary branch.

Chromosomal mapping showed that HAK/KUP/KT genes were distributed across multiple chromosomes of both the A and C sub-genomes (Figure S1). In this visualization, lines connect genes assigned to the same OrthoFinder orthogroup and therefore indicate shared orthogroup membership rather than syntenic relationships. The map provides a descriptive overview of the annotated chromosomal positions and accession representation of HAK/KUP/KT orthogroup members. Because whole-genome collinearity and conserved flanking-gene analyses were not performed, the observed link patterns were not interpreted as evidence of conserved genomic positions or specific gene loss, duplication or expansion events.

**Figure 3:**
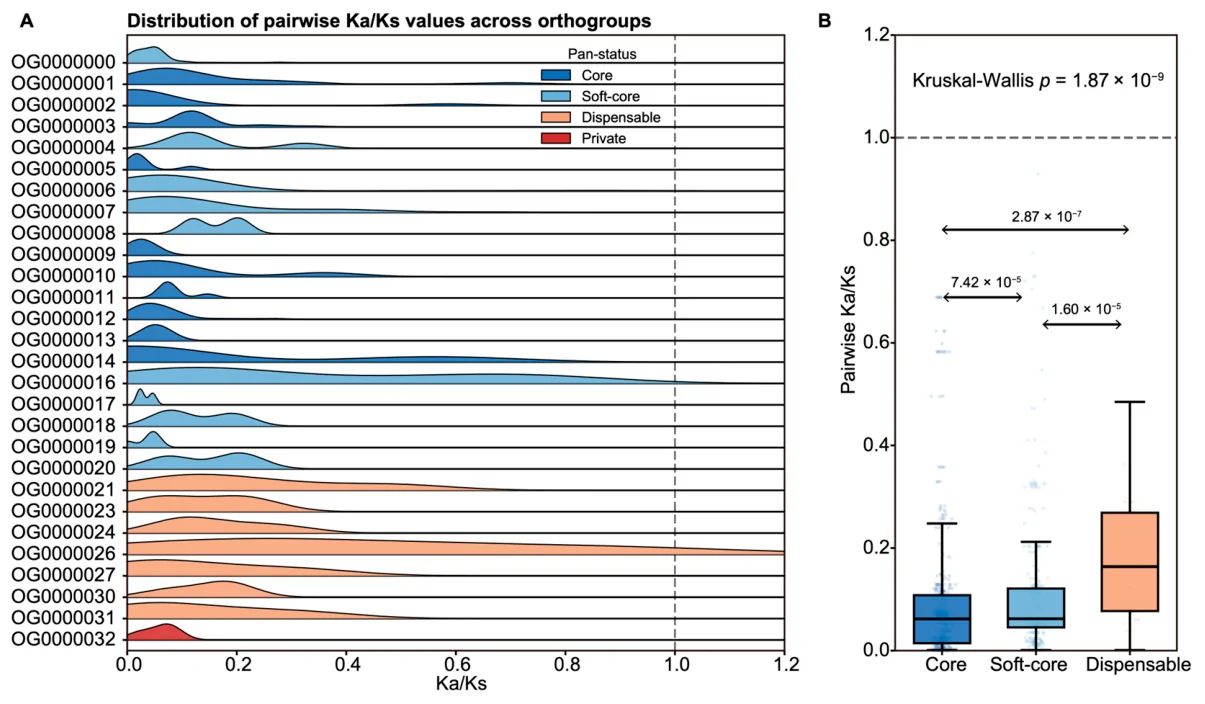
Evolutionary constraint analysis of HAK/KUP/KT orthogroups in *B. napus*. (**A**) Distribution of pairwise *Ka/Ks* values within HAK/KUP/KT orthogroups. Each ridge represents one orthogroup, and colors indicate pan-family categories, including core, soft-core, dispensable and private groups. The vertical dashed line indicates *Ka/Ks* = 1. Most pairwise *Ka/Ks* values were below 1, suggesting predominant purifying selection within the HAK/KUP/KT family. (**B**) Descriptive distribution of valid pairwise Ka/Ks estimates according to the pan-family category of the corresponding orthogroup. Individual pairwise estimates are not statistically independent because the same gene may contribute to multiple comparisons and orthogroups contribute unequal numbers of pairs. The panel is therefore presented as a descriptive visualization only, and no inferential comparison among pan-status categories was performed.

Pairwise *Ka/Ks* analysis showed that most pairwise *Ka/Ks* values within *HAK/KUP/KT* orthogroups were below 1, indicating that this family has mainly evolved under purifying selection (Figure 3A). Descriptive comparison of the pairwise estimates showed that Ka/Ks values assigned to dispensable orthogroups were generally more variable and tended to extend to higher values than those assigned to core and soft-core orthogroups (Figure 3B). However, because pairwise comparisons within an orthogroup are non-independent and orthogroups differ in the number of contributing gene pairs, this pattern was not subjected to formal group-level significance testing and should not be interpreted as direct evidence of relaxed selection. In contrast, the lower Ka/Ks values observed for core orthogroups support comparatively stronger evolutionary constraint among orthogroups represented in all eight accessions.

Overall, these results indicate that HAK/KUP/KT orthogroups differ in both their representation across the eight accessions and their degree of sequence conservation. Core orthogroups showed comparatively stronger evolutionary constraint, whereas orthogroups represented in fewer accessions contributed to the observed annotation-based variation in family composition.

### 3.3. Structural Features and Promoter Cis-Element Variation in HAK/KUP/KT Genes

To examine annotated structural variation within the HAK/KUP/KT family, selected multi-member orthogroups from the core, soft-core and dispensable categories were compared. These orthogroups were used as illustrative examples and were not intended to represent a statistically unbiased sample of the entire family. Members within several orthogroups, including OG0000004, OG0000005 and OG0000022, generally showed similar exon–intron organization, protein length and motif arrangement, indicating conservation of their annotated structures.

Substantial differences in annotated gene and protein lengths were nevertheless observed in several orthogroups. For example, members of OG0000007 ranged from approximately 3.3 kb to more than 9 kb in genomic length and from 607 aa to more than 1200 aa in predicted protein length. Differences in annotated length were also observed in OG0000001 and OG0000025. Because unusually long or short predicted proteins may result from genuine sequence variation, gene model fusion, fragmentation or incomplete annotation, these differences were not interpreted by themselves as evidence of evolutionary structural divergence.

**Figure 4:**
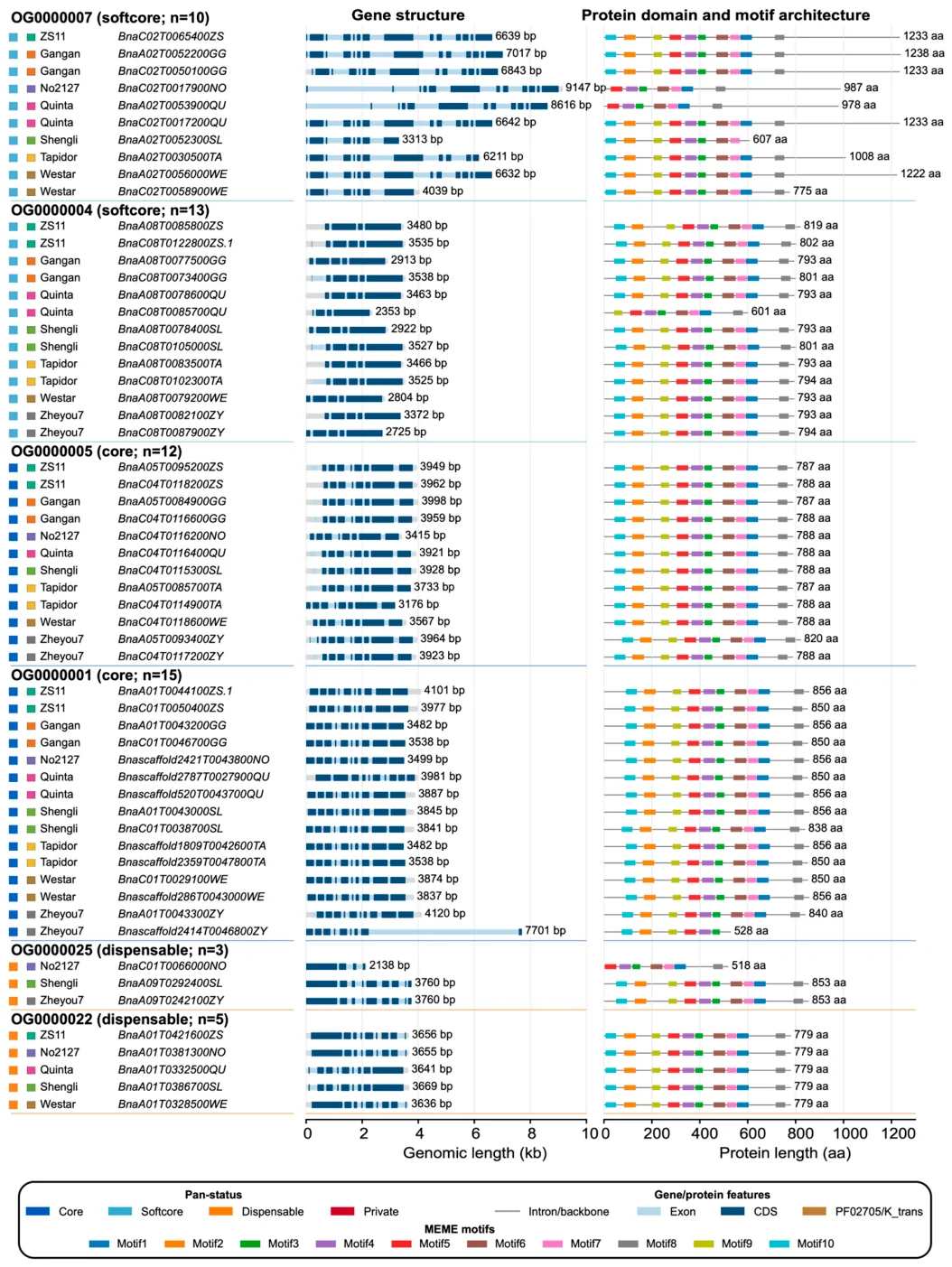
Annotated gene structures, K_trans domain positions and MEME motif patterns of selected HAK/KUP/KT orthogroups in *B. napus*. Multi-member HAK/KUP/KT orthogroups from the core, soft-core and dispensable categories were selected as illustrative examples for comparison of annotated structures across accessions. The left annotation tracks indicate orthogroup membership and the pan-family category assigned to each orthogroup. The middle panel shows exon–intron organization based on genomic coordinates, and the right panel shows the PF02705/K_trans domain and MEME motif organization of the corresponding proteins. Differences in gene length, protein length and motif arrangement are presented descriptively. Unusually long or short predicted proteins may reflect genuine sequence variation, gene model fusion, fragmentation or incomplete annotation and were therefore interpreted cautiously. DeepTMHMM-predicted transmembrane topology for all 269 proteins is provided in Supplementary Table S8.

DeepTMHMM analysis showed that most identified HAK/KUP/KT proteins retained a multi-pass membrane topology. Of the 269 proteins, 230 (85.5%) were predicted to contain 12 transmembrane helices, and 250 (92.9%) contained 10–12 predicted transmembrane helices. However, six proteins were longer than 1000 amino acids, 14 were shorter than 600 amino acids, and several atypical models differed from the predominant 12-helix topology. These predictions provide additional information on protein topology but do not conclusively verify the accuracy of the underlying gene models. All proteins displayed in Figure 4 contained the PF02705/K_trans domain, as expected from the initial family identification procedure; its presence was therefore not considered an independent validation of the unusually long or short gene models. MEME analysis showed that many members within the same orthogroup shared broadly similar motif composition and order. Differences in motif number or arrangement were also observed, particularly in several unusually long or short predicted proteins. Because these differences may result from gene model fusion, fragmentation or incomplete annotation, they were not interpreted as evidence of altered protein architecture or evolutionary structural divergence.

Promoter cis-element analysis revealed variation in the composition of predicted promoter motifs among HAK/KUP/KT genes. Across all identified members, light-related elements were the most abundant category, with 3451 elements detected, followed by stress-related elements with 2360 elements (Figure S2A). JA- and ABA-responsive elements were also frequently detected, with 904 and 848 elements, respectively, whereas auxin-, GA- and development-related elements were less abundant. At the pan-status level, all groups contained multiple types of cis-elements, but their average element counts differed among categories. Descriptive differences in mean predicted motif counts were observed among pan-status categories, with genes belonging to soft-core, dispensable and private orthogroups showing higher mean counts for some motif categories than genes belonging to core orthogroups (Figure S2B). Because no inferential comparison or genome-wide background enrichment analysis was performed, these differences were not interpreted as evidence of greater regulatory activity or regulatory potential.

Orthogroup-level comparison further showed that promoter cis-element composition varied substantially among selected orthogroups (Figure S2C). Several dispensable orthogroups, such as OG0000024 and OG0000035 contained relatively high mean numbers of predicted stress-related elements, whereas OG0000005, OG0000007 and OG0000030 contained relatively high mean numbers of predicted light-related elements. These differences show that predicted promoter cis-element composition varies among multiple pan-family categories and HAK/KUP/KT orthogroups.

Together, these analyses indicate that HAK/KUP/KT genes in *B. napus* retain a conserved transporter-related protein framework while differing in gene structure and predicted promoter cis-element composition. The predicted promoter motifs provide hypotheses regarding possible promoter-level regulation, but their functional activity and contribution to expression differences require experimental validation.

### 3.4. Expression Variation and Candidate Prioritization of Zs11 HAK/KUP/KT Genes

To further explore the potential functional differentiation of *HAK/KUP/KT* genes in the reference accession ZS11, expression profiles of the 41 ZS11 members were analyzed using the BnIR ZS11 expression dataset. Because the BnIR expression profiles use G-type ZS11 gene identifiers, these IDs were matched to the corresponding T-type identifiers used in the annotation and downstream candidate analysis. Tissue expression analysis revealed distinct relative expression patterns across vegetative and reproductive tissues, including floral organs, cotyledons, rosette tissues, leaves at different developmental stages, roots, developing seeds, siliques and stem peels (Figure S3). Some genes showed relatively high expression across multiple tissues, whereas others displayed more tissue-biased patterns, suggesting that ZS11 HAK/KUP/KT members may contribute to potassium transport or homeostasis in different developmental contexts.

**Figure 5:**
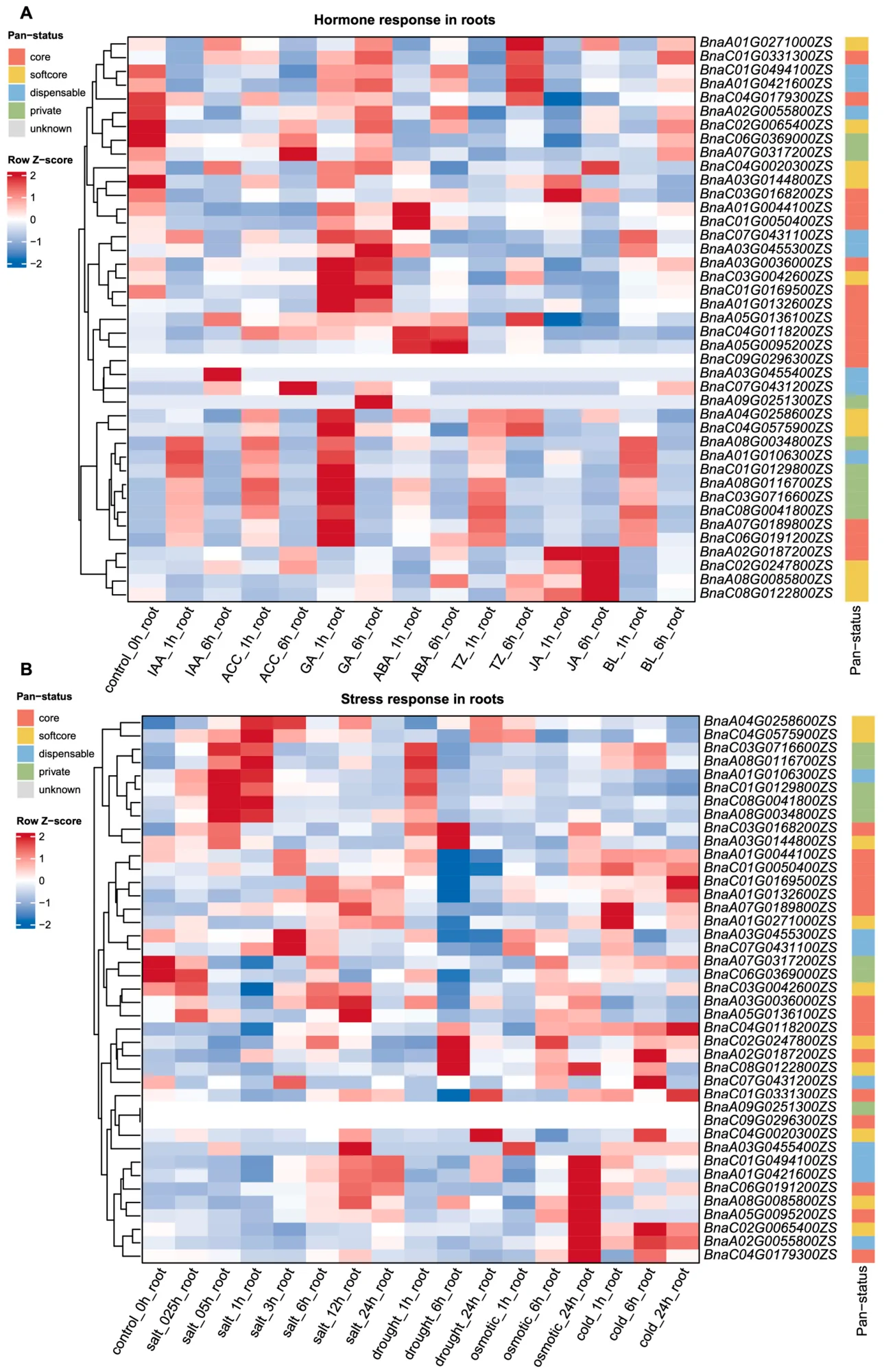
Expression patterns of ZS11 *HAK/KUP/KT* genes under hormone and abiotic stress treatments. (**A**) Heatmap showing relative expression patterns of ZS11 HAK/KUP/KT genes under hormone treatments. (**B**) Heatmap showing relative expression patterns under abiotic stress treatments. Expression values were normalized using row-wise Z-score transformation. Red indicates relatively higher expression, and blue indicates relatively lower expression for each gene. The colors represent relative expression patterns within each gene and do not indicate absolute expression differences among genes or statistically significant differential expression. Side annotation bars represent orthogroup membership and the pan-family category of the orthogroup to which each gene belongs.

Hormone- and stress-treatment expression profiles further showed that ZS11 *HAK/KUP/KT* genes differed in their response patterns in roots (Figure 5). Under hormone treatments, subsets of genes showed altered relative expression after IAA, ACC, GA, ABA, TZ, JA or BL treatment, with responses varying between 1 h and 6 h time points. Under stress treatments, several genes showed relatively higher standardized expression values under salt, drought, osmotic or cold treatment at specific time points. These patterns describe relative expression variation within the BnIR ZS11 dataset and were not interpreted as statistically significant induction. Stress-, light-, JA- and ABA-related motifs were also frequently predicted in the promoter analysis. However, the co-occurrence of these motifs and treatment-responsive expression patterns does not establish a direct regulatory relationship.

To prioritize ZS11 HAK/KUP/KT genes for further investigation, an exploratory integrated scoring analysis was performed by combining expression responsiveness, predicted promoter cis-element composition, pan-status category, orthogroup conservation and phylogenetic information. Predicted promoter features were treated as ancillary sequence information and were not considered independent evidence of regulatory activity. This analysis separated a focused set of high-priority candidates from the 41 ZS11 *HAK/KUP/KT* genes, as summarized in Supplementary Table S5. Among the prioritized genes, *BnaA08T0085800ZS*, *BnaA04T0258600ZS*, *BnaA01T0271000ZS* and *BnaA02T0187200ZS* ranked highly based on multiple descriptive features, including protein characteristics, orthogroup membership, expression patterns and predicted promoter cis-element composition.

*BnaA08T0085800ZS* ranked highly in the exploratory prioritization based on its expression profile, predicted promoter features and evolutionary characteristics and was therefore selected for subsequent integration with published salt-related datasets. Thus, the expression and candidate scoring analyses narrowed the ZS11 *HAK/KUP/KT* family from a genome-wide set of 41 genes to a smaller group of prioritized candidates, providing a basis for integration with published salt GWAS and BnVIR haplotype evidence.

### 3.5. Published Gwas Proximity and Bnvir Haplotype Data Support Exploratory Prioritization of the Bnaa08t0085800zs Locus

**Figure 6:**
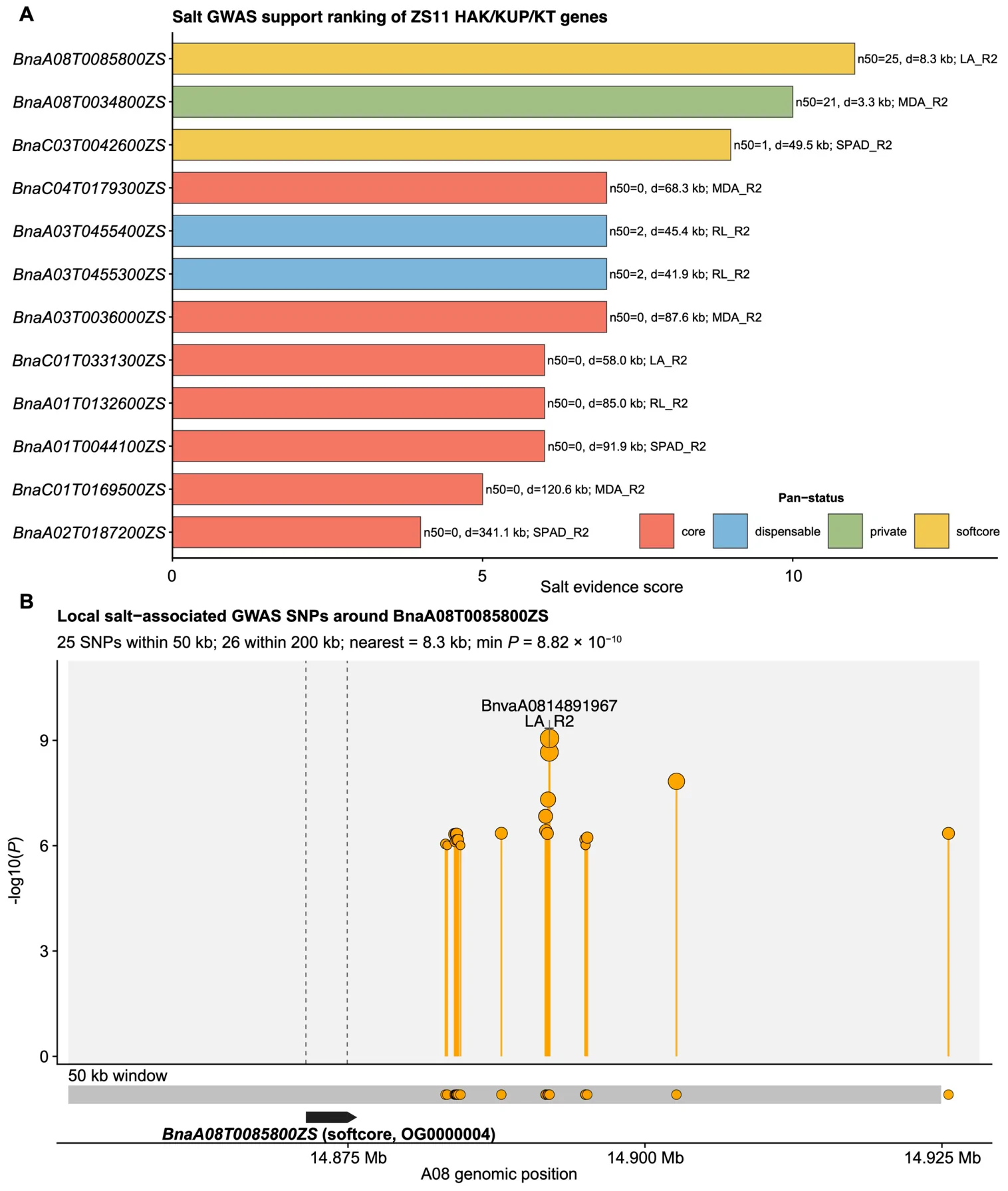
Published salt GWAS proximity of prioritized ZS11 HAK/KUP/KT genes. (**A**) Published GWAS context of prioritized ZS11 HAK/KUP/KT genes. Candidate genes are shown according to their locus-level GWAS context based on nearby salt-associated SNP information. *BnaA08T0085800ZS* showed the strongest locus-level GWAS context among the analyzed candidates. (**B**) Local distribution of salt-associated GWAS SNPs around *BnaA08T0085800ZS* on chromosome A08. Each vertical line represents a nearby GWAS SNP, and point height indicates the association strength. The strongest nearby SNP, BnvaA0814891967 [7], was associated with the salt-related leaf area trait LA_R2. The gene model of *BnaA08T0085800ZS* is shown at the bottom, and dashed lines indicate the candidate gene region.

To determine whether the prioritized ZS11 HAK/KUP/KT genes were located near published salt-associated SNPs, published salt GWAS SNPs were integrated with the physical positions of ZS11 *HAK/KUP/KT* genes. Among the analyzed candidates, *BnaA08T0085800ZS* showed the strongest locus-level GWAS context among the prioritized candidates (Figure 6A). This gene is located on chromosome A08 and belongs to the soft-core orthogroup OG0000004, which was represented in six or seven of the eight analyzed *B. napus* accessions. Phylogenetic analysis placed *BnaA08T0085800ZS* within a branch containing *AtKUP10* and several rice HAK/KUP/KT reference proteins, including *OsHAK11* and *OsHAK12* (Figure 2). This placement was used only to describe its phylogenetic context and was not interpreted as evidence of one-to-one orthology or functional equivalence.

Local GWAS proximity analysis showed that multiple salt-associated SNPs were located near *BnaA08T0085800ZS*. In total, 25 SNPs were detected within the 50 kb flanking region, and 26 SNPs were detected within the 200 kb window around this gene (Figure 6B). The nearest GWAS SNP was located approximately 8.3 kb from the gene, and the strongest nearby association showed a minimum *p* value of 8.82 × 10^−10^. The strongest associated SNP, BnvaA0814891967 [7], was located downstream of *BnaA08T0085800ZS* and was associated with the salt-related leaf area trait (LA_R2). These results show that *BnaA08T0085800ZS* is located within a genomic interval containing multiple published salt-associated SNPs; however, this proximity does not identify the causal gene or variant within the interval.

**Figure 7:**
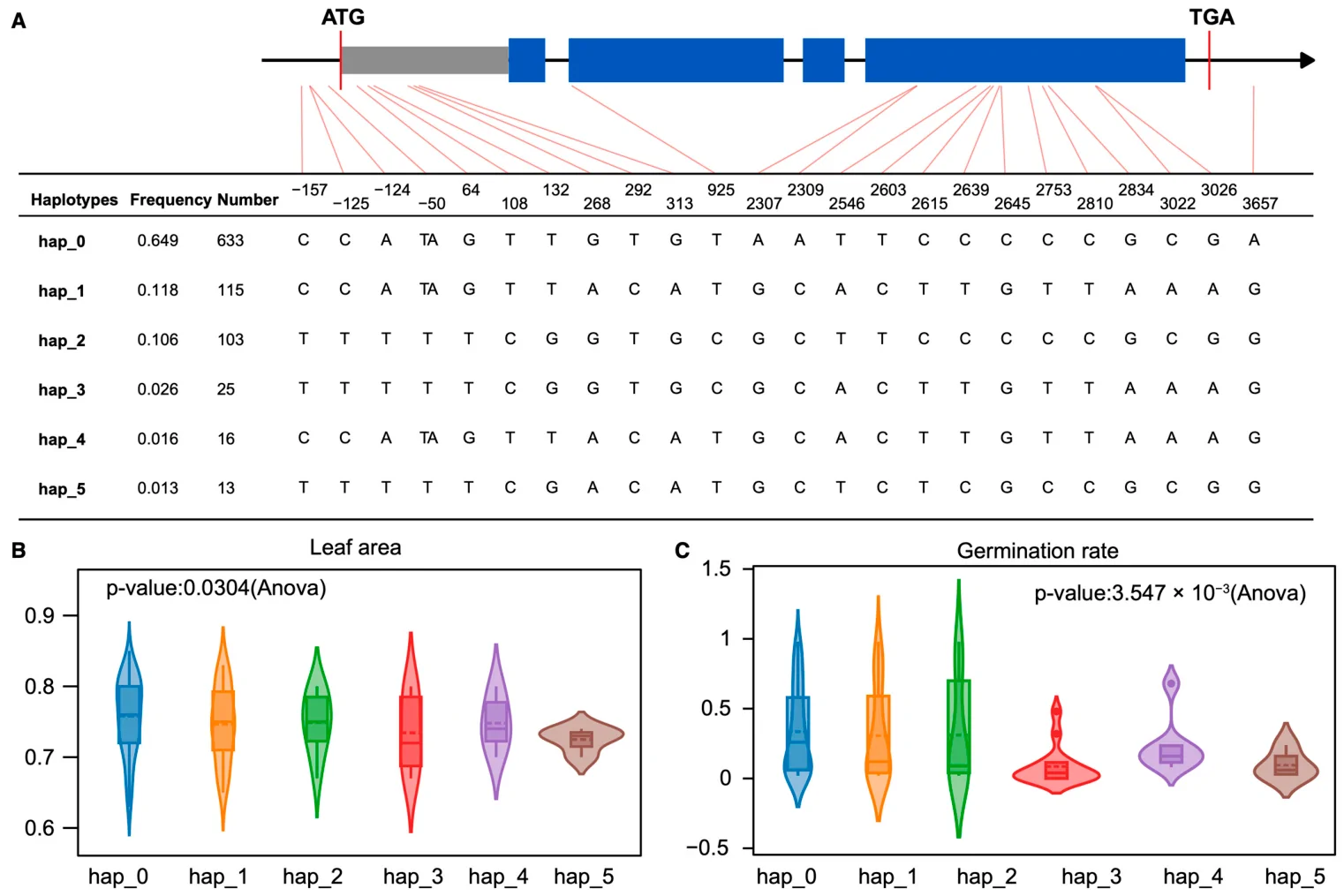
Haplotype–phenotype association analysis of *BnaA08G0085800ZS* using BnVIR. (**A**) Gene structure, SNP distribution and major haplotypes of BnaA08G0085800ZS. Blue boxes represent exons, gray boxes represent untranslated regions, and red lines indicate SNP positions. (**B**,**C**) Violin plots showing the phenotypic distributions of leaf area (**B**) and germination rate (**C**) among major haplotypes. The observed haplotype-associated phenotypic differences provide additional locus-level information for candidate prioritization but do not demonstrate a causal effect of *BnaA08G0085800ZS*. Haplotype group sizes were unequal, and the displayed database-reported *p* values were not adjusted for population structure, kinship, environmental effects or multiple testing. The plots are therefore presented descriptively and do not establish a causal or statistically robust haplotype effect.

To obtain additional population-level information, *BnaA08T0085800ZS* was matched to the corresponding BnVIR identifier *BnaA08G0085800ZS*. BnVIR records showed that this locus contains multiple SNPs that define several haplotypes in the natural population. In the displayed haplotype matrix, six major haplotypes were shown, among which hap_0 was the most frequent haplotype, with a frequency of 0.649 and 633 accessions, followed by hap_1 and hap_2, with frequencies of 0.118 and 0.106, respectively (Figure 7A). The remaining haplotypes occurred at lower frequencies.

BnVIR displayed differences in the phenotype distributions among the six haplotype groups, although the group sizes were highly unequal. Nominal ANOVA *p* values reported by the database were 0.0304 for leaf area (Figure 7B) and 3.547 × 10^−3^ for germination rate (Figure 7C). However, these values were not adjusted for population structure, relatedness, environmental variation or multiple testing and were therefore not interpreted as statistically robust haplotype effects. The BnVIR results are presented only as descriptive locus-level information for candidate prioritization. Consistent with its prioritization as a salt-related candidate, qRT-PCR analysis showed that BnaA08T0085800ZS was responsive to 200 mM NaCl treatment, with the highest relative expression observed at 6 h (Figure S4).

Taken together, the salt-responsive expression profile, published GWAS proximity and BnVIR haplotype–phenotype information provide complementary but indirect evidence for prioritizing the *BnaA08T0085800ZS* locus as a HAK/KUP/KT candidate associated with salt-related variation. These analyses do not establish that *BnaA08T0085800ZS* itself causally underlies the associated phenotypes, because the observed signals may reflect variation in the gene, its regulatory region or another linked locus.

## 4. Discussion

*HAK/KUP/KT* transporters play important roles in plant potassium uptake, translocation and ion homeostasis, and have also been implicated in plant development and stress responses [8]. In *B. napus*, previous studies have characterized the *HAK/KUP/KT* family mainly using a single reference genome and suggested potential roles of several members in potassium-deficiency responses [16]. However, the extensive presence–absence variation and genome diversification revealed by the *B. napus* pan-genome indicate that single-genome analyses may not fully capture accession-level gene family diversity [17]. In this study, we performed a pan-family analysis of *HAK/KUP/KT* genes across eight *B. napus* accessions, which allowed conserved and accession-variable members of this transporter family to be distinguished at the orthogroup level. By integrating phylogenetic classification, chromosomal distribution of orthogroup members, Ka/Ks analysis, gene structure, promoter cis-elements and expression profiles, we further evaluated evolutionary conservation and variation in gene structure, predicted promoter features and expression profiles within this family. Finally, published salt GWAS signals [7] and BnVIR [31] haplotype evidence were incorporated to prioritize candidate genes associated with salt-related variation, highlighting *BnaA08T0085800ZS* as a candidate prioritized for further investigation in future functional studies.

### 4.1. Pan-Family Analysis Expands the View of HAK/KUP/KT Diversity in B. napus

Single reference gene family analysis is useful for initial gene identification, but it may underestimate accession-level gene content variation, particularly in polyploid crops. Recent pan-genome-based studies in *B. napus* have shown that gene families can display substantial copy number variation, presence–absence variation and accession-specific distribution. Pan-family studies of SPL [18], SWEET [19] and transporter gene families [20] have similarly demonstrated that multi-genome resources can reveal gene content variation that is not captured by a single reference genome.

Compared with the previous genome-wide survey of the *HAK/KUP/KT* family in *B. napus*, our analysis provides a different view of this transporter family. The previous single reference analysis established the basic classification of the BnaHAK family [16], whereas the present pan-family framework shows that its gene composition is not fixed across accessions. In contrast, our pan-family analysis groups HAK/KUP/KT members into orthogroups that are broadly or more restrictively represented across the eight published genome annotations. A similar classification has been applied to the R2R3-MYB and TPS families in *B. napus*, in which conserved and variable components differed in sequence evolution, predicted promoter features or abiotic stress expression patterns [33,34]. Therefore, pan-family analysis is not merely an expansion in gene numbers, but provides a framework for interpreting gene family conservation, sequence divergence and accession-level variation.

This distinction is particularly relevant in *B. napus*, where polyploid genome evolution can generate gene presence–absence variation and copy number variation. Previous pan-genome analysis showed that homoeologous exchange is a major cause of gene PAV in amphidiploid *B. napus*, and a substantial proportion of variable genes are predicted to be involved in agronomically important processes [35]. Similarly, copy number variation has been reported in resistance gene analogs across eight *B. napus* lines, indicating that accession-level variation can substantially alter the composition of multi-copy gene families [36]. In the present study, dispensable and private HAK/KUP/KT orthogroups represent orthogroups detected in subsets of the eight published genome annotations, whereas core and soft-core orthogroups were represented more broadly across the analyzed accessions. The pairwise Ka/Ks distributions varied among orthogroups and pan-status categories; however, these descriptive patterns were not used to infer differences in selection pressure because the contributing pairwise estimates were non-independent and represented heterogeneous evolutionary relationships. However, these annotation-based representation patterns do not by themselves establish specific gene loss, duplication or accession-specific expansion events, nor do they demonstrate essential physiological functions or adaptive specialization. The results should therefore be interpreted as evidence that the HAK/KUP/KT family comprises broadly represented and more restrictively represented components with different degrees of sequence conservation.

### 4.2. Evolutionary Constraint and Putative Regulatory Variation in the HAK/KUP/KT Family

The predominance of Ka/Ks values below 1, together with the retention of the PF02705/K_trans domain, suggests that the protein framework of the *B. napus* HAK/KUP/KT family has remained under evolutionary constraint. This is consistent with recent functional studies showing that *HAK/KUP/KT* members can act as high-affinity K^+^ transport systems. For example, *AtHAK5* was recently demonstrated to function as a proton-motive-force-powered high-affinity K^+^ transporter in *Arabidopsis*, while mechanistic studies have shown that specific HAK5 domains and phosphorylation-dependent regulation are important for its transport activity [37,38]. These findings provide family-level context for interpreting the conserved domain and motif framework observed in *B. napus*, which is consistent with evolutionary constraint on transporter-related protein features.

However, conservation at the protein level does not imply that all family members have identical biological roles. Differences in gene structure, predicted promoter cis-element composition and expression patterns provide descriptive evidence of variation among orthogroups, but they do not demonstrate the regulatory mechanisms responsible for these differences. More broadly, plant K^+^ transport systems are regulated at multiple levels, including nutrient sensing and signaling networks. For instance, potassium nutrient status can drive post-translational regulation of K^+^ channels and carriers, including HAK5-related pathways, through CBL–CIPK signaling [39]. Accordingly, the expression differences observed among ZS11 HAK/KUP/KT genes cannot be directly attributed to the predicted promoter motifs, whose regulatory activity requires experimental validation.

Functional studies in other plant species illustrate that members of the structurally conserved HAK/KUP/KT family can nevertheless perform distinct roles in ion transport and stress responses. In rice, *OsHAK1* contributes to K^+^ uptake and salt tolerance under both low- and high-K^+^ conditions, while *OsHAK21* is involved in maintaining Na^+^/K^+^ homeostasis and salt stress tolerance [40,41]. These examples demonstrate functional diversity within the broader HAK/KUP/KT family, but they do not establish equivalent roles for the *B. napus* genes analyzed here. The evolutionary and expression analyses in this study therefore provide a basis for prioritizing *B. napus* HAK/KUP/KT members for further functional investigation rather than assigning specific stress-response functions.

### 4.3. Bnaa08t0085800zs Is a Prioritized Candidate Locus near Salt-Associated Variation

Published GWAS context and BnVIR haplotype–phenotype information were considered as exploratory locus-level evidence when prioritizing *BnaA08T0085800ZS*. Phylogenetically, *BnaA08T0085800ZS* was placed within a branch containing *AtKUP10*, *OsHAK11* and *OsHAK12*. However, reciprocal sequence-similarity analysis, conserved synteny and the relationships among corresponding genes in *B. rapa*, *B. oleracea* and the A- and C sub-genomes of *B. napus* were not examined. We therefore use this placement only to describe the phylogenetic context of *BnaA08T0085800ZS* and do not designate it as an *AtKUP10-like* gene or a direct *AtKUP10* ortholog. *OsHAK11* and *OsHAK12* were included as reference members for clade-level phylogenetic interpretation [9]. However, phylogenetic proximity to *OsHAK12* does not establish functional equivalence, and the experimentally characterized salt-related function of *OsHAK12* cannot be transferred to *BnaA08T0085800ZS* on the basis of their placement in the same broad phylogenetic branch.

The BnVIR haplotype–phenotype information provided additional locus-level context for *BnaA08G0085800ZS*. Differences in leaf area and germination rate were observed among the major haplotype groups. However, the haplotype groups were highly unequal in size, and the database-derived comparisons did not account for population structure, relatedness, environmental variation or multiple testing. These patterns should therefore be regarded as exploratory and may reflect confounding factors rather than direct effects of the *BnaA08G0085800ZS* haplotypes. In addition, because the extent of accession and phenotype overlap between BnVIR and the published GWAS dataset was not established, the GWAS and BnVIR results were not treated as independent evidence. The nearby GWAS signals may affect BnaA08T0085800ZS itself, its regulatory region or another linked gene in the same genomic interval. Future work should determine the transport activity and physiological function of BnaA08T0085800ZS and test whether different haplotypes show distinct expression, K^+^/Na^+^ accumulation or salt tolerance phenotypes.

## 5. Conclusions

In this study, we performed a pan-family analysis of *HAK/KUP/KT* potassium transporter genes across eight *B. napus* accessions and identified 269 annotated family members. Orthogroup classification revealed both conserved and accession-variable components, including core, soft-core, dispensable and private HAK/KUP/KT orthogroups. Phylogenetic, Ka/Ks and structural analyses indicated conservation of the transporter-related protein framework, whereas differences were observed in gene structure, predicted promoter cis-element composition and expression profiles among orthogroups. Expression profiling further indicated that ZS11 *HAK/KUP/KT* genes exhibit tissue-, hormone- and stress-responsive variation. By integrating salt-associated expression patterns, published GWAS context and BnVIR haplotype–phenotype information, *BnaA08T0085800ZS* was prioritized as a HAK/KUP/KT candidate locus near published salt-associated variation. Overall, this study expands the understanding of HAK/KUP/KT family diversity in *B. napus* and provides a prioritized candidate for future functional testing of potassium transport, ion homeostasis and salt-response hypotheses.

## Data Availability

The datasets used in this study are publicly available from the cited sources, and all derived data and scripts are available from the corresponding author upon reasonable request.

## Supplementary Materials

The following supporting information can be downloaded at: https://www.mdpi.com/article/10.3390/genes17080893/s1, Figure S1: Chromosomal distribution and orthogroup membership of HAK/KUP/KT genes across eight Brassica napus accessions; Figure S2. Promoter cis-element composition of HAK/KUP/KT genes in B. napus; Figure S3. Tissue-specific expression patterns of ZS11 HAK/KUP/KT genes; Figure S4. Salt-induced expression pattern of BnaA08T0085800ZS under 200 mM NaCl treatment; Table S1: Identification and basic annotation of 269 HAK/KUP/KT family members across eight *B. napus* accessions; Table S2: Orthogroup membership, accession-level copy numbers, and pan-family classification of HAK/KUP/KT family members; Table S3: Gene structure, K_trans domain, and MEME motif features of selected HAK/KUP/KT orthogroups; Table S4: Predicted promoter cis-element sites, gene-level counts, and summaries by pan-status and orthogroup; Table S5: ZS11 tissue, hormone, and abiotic-stress expression profiles, derived expression features, and integrated candidate-prioritization results; Table S6: Published salt-associated GWAS SNPs and their proximity to ZS11 *HAK/KUP/KT* genes; Table S7: *A. thaliana* and *O. sativa* reference HAK/KUP/KT proteins used for phylogenetic analysis; Table S8: DeepTMHMM-predicted transmembrane topology of 269 HAK/KUP/KT proteins.
