# Supplementary material for: Pan-Family Analysis of HAK/KUP/KT Potassium Transporters in Brassica napus Prioritizes a Candidate Locus Associated with Salt-Related Variation
Source: Genes (Basel). 2026 Jul 29;17(8):893. doi: 10.3390/genes17080893 (PMC13512543; doi:10.3390/genes17080893)
Supplement: Supplementary file 1 [file genes-17-00893-s001.zip › Supplementary file.pdf]

Supplementary Figures：

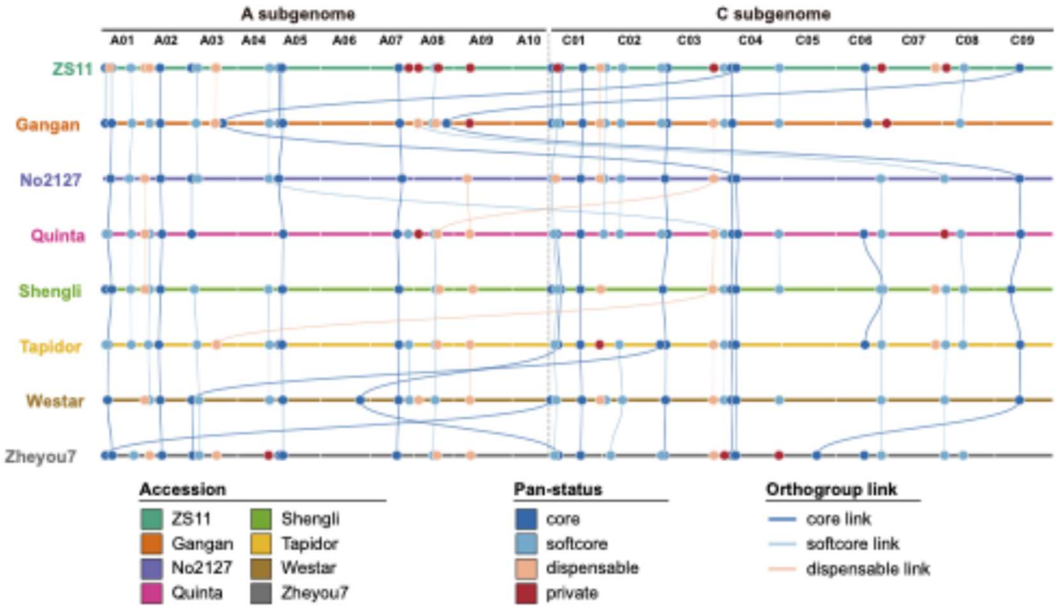

Figure S1. Chromosomal distribution and orthogroup membership of HAK/KUP/KT genes across eight *Brassica napus* accessions. HAK/KUP/KT genes identified from ZS11, Gangan, No2127, Quinta, Shengli, Tapidor, Westar and Zheyou7 are displayed on chromosomes of the A and C subgenomes according to their annotated genomic positions. Colored dots represent individual genes and indicate the pan-family category of the orthogroup to which each gene belongs, including core, soft-core, dispensable and private orthogroups. Lines connect genes assigned to the same OrthoFinder orthogroup and indicate shared orthogroup membership only. These links do not represent synteny, conserved genomic positions, or specific gene loss, duplication or expansion events. The figure provides a descriptive overview of the annotated chromosomal distribution and accession representation of HAK/KUP/KT orthogroup members.

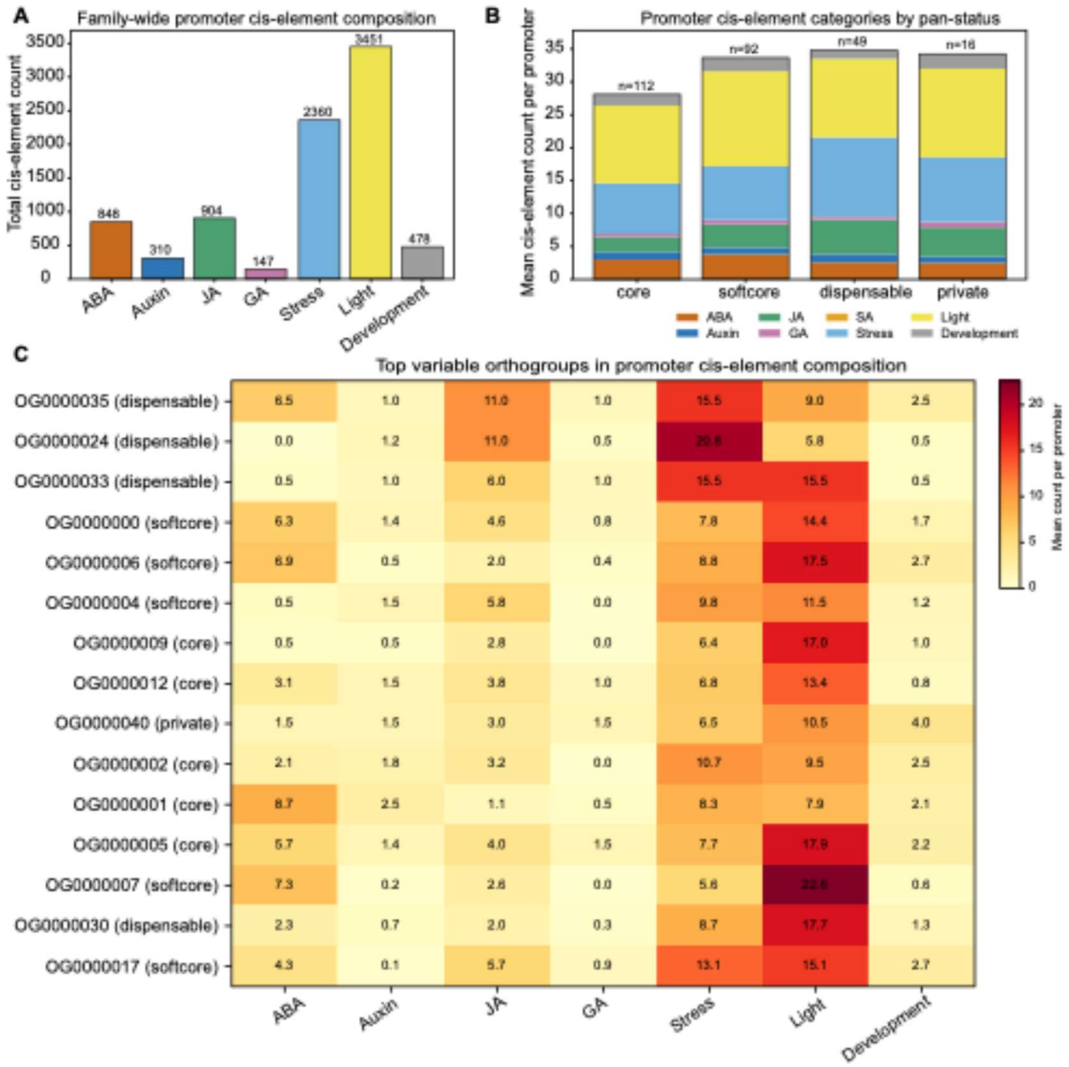

Figure S2. Promoter cis-element composition of HAK/KUP/KT genes in *B. napus*. (A) Total numbers of different types of cis-acting regulatory elements identified in the 2-kb upstream promoter regions of genes. Elements were grouped into functional categories, including hormone-, stress-, light- and development-related elements. (B) Average promoter cis-element composition of HAK/KUP/KT genes from different pan-family categories, including core, soft-core, dispensable and private groups. (C) Heatmap showing the average number of major cis-element categories in selected HAK/KUP/KT orthogroups. Color intensity represents the mean number of cis-elements in each category. The results indicate that *HAK/KUP/KT* genes contain diverse promoter regulatory elements, with orthogroup- and pan-status-associated differences in cis-element composition.

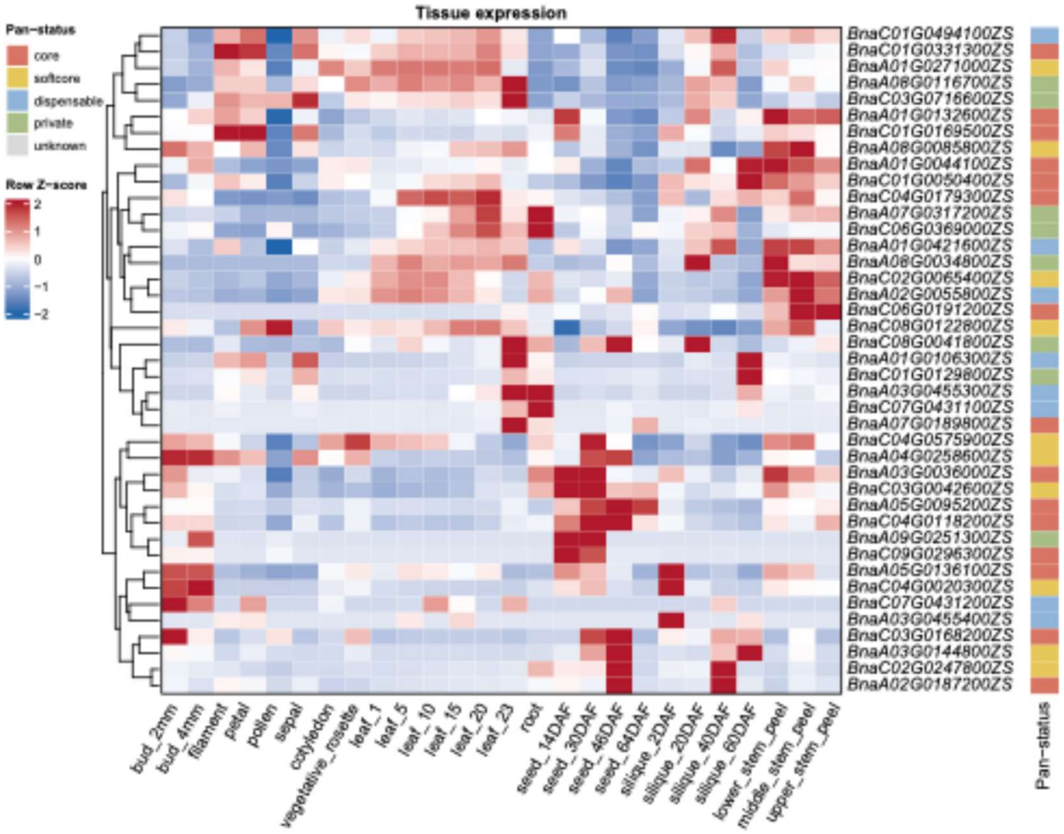

Figure S3. Tissue-specific expression patterns of ZS11 *HAK/KUP/KT* genes. Relative expression profiles of ZS11 HAK/KUP/KT genes across multiple tissues and developmental stages were visualized using the BnIR ZS11 expression dataset. Expression values were normalized by row-wise Z-score transformation. Red represents relatively higher expression and blue represents relatively lower expression for each gene. Side annotation bars indicate pan-family category and orthogroup information.

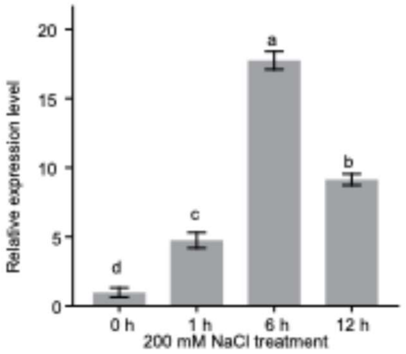

Figure S4. Salt-induced expression pattern of *BnaA08T0085800ZS* under 200 mM NaCl treatment. Relative expression levels of *BnaA08T0085800ZS* were examined by qRT-PCR at 0, 1, 6 and 12 h after 200 mM NaCl treatment. Expression levels were normalized to the internal reference gene and calculated using the $2^{-\Delta\Delta Ct}$ method, with the 0 h sample set as the control. Data are shown as mean ± SD of four biological replicates. Different letters indicate significant differences among time points.

Supplementary Table：

Supplementary Table S1. Genome-wide identification and basic annotation of HAK/KUP/KT genes in eight Brassica napus genomes

Supplementary Table S2. Orthogroup clustering and pan-family classification of HAK/KUP/KT genes in eight Brassica napus genomes.

Supplementary Table S3. Gene structure, K_trans domain and MEME motif features of selected HAK/KUP/KT orthogroups.

Supplementary Table S4. Promoter cis-acting element analysis of HAK/KUP/KT genes.

Supplementary Table S5. Expression features and integrated candidate scoring of ZS11 HAK/KUP/KT genes.

Supplementary Table S6. Published salt GWAS SNP proximity analysis for ZS11 HAK/KUP/KT genes.
